# Sodium–Glucose Cotransporter-2 Inhibitors After Acute Myocardial Infarction

**DOI:** 10.3390/biomedicines13030720

**Published:** 2025-03-15

**Authors:** Nicia I. Profili, Roberto Castelli, Roberto Manetti, Marta C. Sircana, Michela Pagni, Gemma Lisa Sechi, Antonio Gidaro, Costantino Cossu, Francesco Bella, Alessandro P. Delitala

**Affiliations:** 1 Department of Medicine, Surgery, and Pharmacy, University of Sassari, 07100 Sassari, Italyrmanetti@uniss.it (R.M.); m.pagni@studenti.uniss.it (M.P.);; 2 Department of Biomedical and Clinical Sciences Luigi Sacco, University of Milan, Luigi Sacco Hospital, 20157 Milan, Italy; 3 Azienda Ospedaliero-Universitaria di Sassari, 07100 Sassari, Italyfrancesco.bella@aouss.it (F.B.)

**Keywords:** SGLT2i, sodium–glucose cotransporter-2 inhibitors, acute myocardial infarction, acute heart failure, diabetes, cardiovascular disease

## Abstract

Sodium–glucose cotransporter-2 inhibitors (SGLT2i) are a specific class of drugs originally developed for treating type 2 diabetes mellitus. Subsequently, studies demonstrated that their action was not limited to glycemic control but could also have positive effects on other specific outcomes, particularly at the cardiovascular level. Indeed, due to their diuretic effect, SGLT2i improve the clinical control of chronic heart failure and reduce the risk of rehospitalization. In addition, other studies reported a protective effect on major cardiovascular events and mortality. More recently, it has been suggested that the prescription of SGLT2i after an acute myocardial infarction may have positive effects due to their possible effect on inflammation, arrhythmias, and ventricular remodeling. Here, we reviewed studies focused on SGLT2i after an acute myocardial infarction in patients treated with percutaneous coronary intervention.

## 1. Introduction

Acute myocardial infarction (AMI) is defined as cardiomyocyte necrosis in the clinical setting of acute myocardial ischemia [1]. Guidelines suggested the use of the term AMI in the presence of clinical evidence of acute myocardial ischemia and the detection of a rise and/or fall of high-sensitivity cardiac troponin T associated with at least one of the following criteria: symptoms of myocardial ischemia, new electrocardiogram changes consistent with ischemia, the development of pathological Q waves, the detection of intracoronary thrombus, or imaging evidence of the loss of viable myocardium or new regional wall motion abnormalities [1]. AMI can be classified into different types according to clinical, pathological, and prognostic criteria. The choice of treatment is based on a specific algorithm that can be time-dependent in patients with high-risk features. Percutaneous coronary intervention (PCI) is the preferred reperfusion strategy [2]. Pharmacological treatment has a pivotal role soon after PCI. Indeed, oral anti-platelet agents are promptly prescribed to reduce the risk of local thrombotic complications and systemic ischemic events, while angiotensin-converting enzyme (ACE) inhibitors and β-blockers are useful to improve outcomes in post-AMI patients complicated by heart failure, diabetes, and/or hypertension [2].

Sodium–glucose cotransporter-2 inhibitors (SGLT2i) are a specific class of drugs initially used to treat type 2 diabetes mellitus [3]. Thereafter, it has been demonstrated that in addition to their glycosuric effect, SGLT2i also provide several benefits for cardiovascular and kidney diseases [4]. Indeed, the glycosuric effect led to a diuretic effect, which in turn, reduces intravascular volume and arterial pressure. Thanks to these hemodynamic effects, SGLT2i showed efficacy in the treatment of chronic heart failure. Experimental and clinical data also demonstrated additional mechanisms: SGLT2i improved adipose tissue metabolism, reduced systemic inflammation, increased insulin sensitivity [4], and improved kidney function [5].

The glycosuric effect may be also useful in cases of acute decompensated heart failure. Studies showed how the prescription of SGLT2i, in addition to the conventional treatment, can increase the chance of positive outcomes (i.e., improved patient-reported symptoms and reduced hospitalization for heart failure) [6]. More recently, studies suggested that SGLT2i may have a role in the management of patients with AMI. Specifically, SGLT2i seem to reduce the risk of specific negative outcomes that can develop after AMI. To better understand the role of SGLT2i in these patients, we revised the clinical studies focused on SGLT2i prescribed to patients with AMI treated with PCI.

## 2. Biology of Sodium–Glucose Cotransporter

The sodium–glucose cotransporter (SGLT) plays a critical role in humans due to its activity to drive glucose and sodium ions into cells [7]. SGLTs belong to the solute carrier family 5, which includes 12 different member proteins that mediate the transport of sugars (SGLT1-5), vitamins (SGLT6, SGLT7), myo-inositol (SGLT3 and SGLT11), iodide (SGLT5), choline (SGLT7), and monocarboxylate (SGLT8 e SGLT12) [8].

SGLT1’s function is to absorb glucose and galactose, but not fructose or mannose, across the intestinal brush-border membrane. Compared to other channels, the conductance of SGLT1 for urea and water is relatively low, but the high levels of its expression in enterocytes explain its critical role in the control of the transport of water and urea across the intestinal membrane [7]. Indeed, the genetic disease glucose–galactose malabsorption causes severe diarrhea in the presence of glucose or galactose in the diet. SGLT1 also regulate the reabsorption of these sugars in the region S2 and S3 of the proximal tubules, which occurrs with a stoichiometry of two sodium ions for each sugar molecule [9].

**Figure 1:**
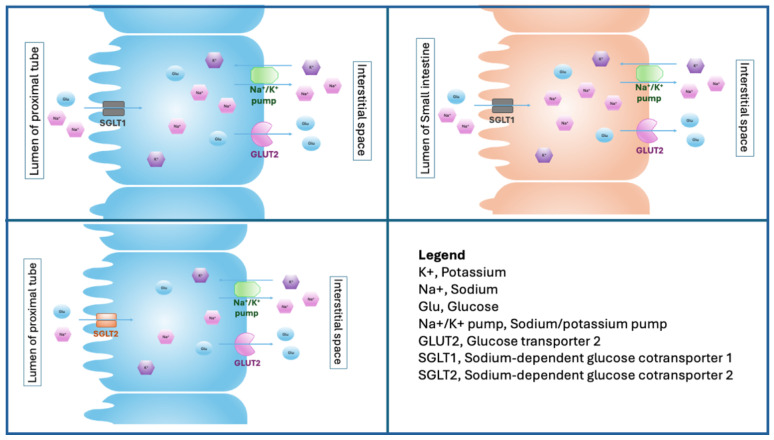
Site and function of SGLT.

SGLT2 channels are located in the proximal renal convoluted tubule and are responsible for reabsorbing approximately 90% of the filtered glucose (Figure 1) [10]. Mutations in the gene encoding SGLT2 are responsible for familial renal glycosuria, a disease characterized by the presence of glycosuria despite normal blood glucose levels [11]. Despite its relatively low affinity, SGLT2 is a high-capacity transporter. The glucose reabsorption is sodium-dependent (1:1 stoichiometry) and, therefore, 4–5% of daily filtered sodium load is reabsorbed [11]. Sodium is pumped out of the epithelial cells into the blood through the sodium/potassium-ATPase. This transepithelial flux generates fluid absorption due to the osmotic gradient. SGLT2 expression in proximal tubes is increased in diabetic patients, but the mechanism of this upregulation has not been fully elucidated [12].

SGLT2i can reduce the renal threshold for glucose excretion from 180 mg/dL to 40 mg/dL, thus increasing glycosuria. This effect, found in diabetics and healthy people, further induces additional effects unrelated to glycemic control: increased diuresis, reduced inflammation, and weight loss [6]. Sodium–glucose cotransporter-2 inhibitors have a different activity on SGLT1 and SGTL2. Indeed, empagliflozin has the selectivity of SGLT2, while canagliflozin has the lowest [13].

## 3. The Effects of SGLT2i on Inflammatory Biomarkers After AMI

Atherosclerosis is a chronic inflammatory disease of the arterial wall. The exact pathogenesis is unclear, but specific cytokines (interleukin 6, vascular cell adhesion molecule 1, and intercellular adhesion molecule 1) are involved in this process [14]. The role of SGLT2i on inflammatory biomarkers after AMI has been evaluated in two main studies with different results. One study reported the effect of SGLT2i on inflammatory burden and infarct size [15]. This multicenter observational registry included 583 diabetic patients who were admitted with ST-segment elevation myocardial infarction (STEMI) and non-ST-segment elevation myocardial infarction (NSTEMI). The patients who underwent PCI were divided into two groups according to the antidiabetic treatment on admission: SGLT2i users (started at least 3 months before) vs. non-SGLT2i users, including those receiving other antidiabetic drugs. The authors compared inflammatory biomarkers and infarct size between the two groups. They found that baseline treatment with SGLT2i was associated with reduced white blood cell count, neutrophils, and C-reactive protein. In addition, the group of SGLT2i users displayed a smaller infarct size, as evidenced by lower troponin values and lower peaks [15]. On the contrary, a post hoc analysis of the EMMY trial compared the mean change in inflammatory biomarkers (high-sensitivity C-reactive protein, IL-6, neutrophils, leukocytes, and their ratios) after 26 weeks of treatment with either the SGLT2i empagliflozin or placebo in patients with AMI treated with PCI [16]. Authors demonstrated a significant mean reduction, from the baseline to the end of the follow-up, for neutrophils (−20.46%, *p* < 0.001), leukocytes (−10.22%, *p* < 0.001), IL-6 (−57.37%, *p* < 0.001), and high-sensitivity C-reactive protein (26.1%, *p* < 0.001) in patients after AMI. However, treatment with empagliflozin showed no impact on this decline, thus indicating no role in blunted systemic inflammation [16].

## 4. The Effects of SGLT2i on Cardiac Sympathetic and Parasympathetic Activity

The autonomic nervous system influences most heart functions at multiple levels: sinoatrial node, atrioventricular node, myocardium, and vessels. Abnormalities of both sympathetic and parasympathetic systems can cause different heart manifestations. Heart rate variability (HRV) represents the physiological phenomenon of beat-to-beat variations in cardiac cycle length. Increased sympathetic or decreased sympathetic activity may lead to an abnormal HRV, which is a strong predictor of mortality and sudden arrythmias in patients with AMI [17]. HRV parameters reflect the autonomic modulation of the heart for a given period and its quantification can be achieved using time–domain, frequency–domain, and nonlinear methods. For the time–domain method, the root mean square of the successive differences (RMSSD) and the standard deviation of the 5 min average NN intervals (SDANN) are the most studied parameters.

RMSSD reflects the beat-to-beat variance in heart rate and is used to estimate the vagally mediated changes reflected in HRV [18], while SDANN shows how far the HRV is from that average at any point in the day. Frequency–domain measurements calculate the amount of signal energy within component bands. The Task Force of the European Society of Cardiology and the North American Society of Pacing and Electrophysiology (1996) divided heart rate oscillations into four frequency bands: ultra low-frequency (ULF), very low-frequency (VLF), low-frequency (LF), and high-frequency (HF) bands [19]. LF and HF are the most commonly used indices, and the latter may reflect the parasympathetic activity of the autonomic nervous system [20].

The EMBODY trial was conceived to evaluate whether the use of SGLT2i was accompanied by an improvement of cardiac nerve activity in patients with AMI and diabetes mellitus [21]. Specifically, the trial assessed the mechanisms by which empagliflozin affected the measurement of HRV, heart rate turbulence (HRT), late potential (LP), and T wave alternans (TWA). The authors randomized 96 diabetic patients to receive either empagliflozin 10 mg/day or placebo (as an add-on to the conventional treatment) 2 weeks after the onset of AMI. After 24 weeks of follow-up, the empagliflozin group showed a significant increase from the baseline SDANN intervals (+11.6 ms, *p* = 0.02) and RMSSD (+6.5 ms, *p* = 0.01). Changes in HF power and sympathovagal balance (LF/HF ratio) were further significant in the treatment group (respectively, +583.1 ms^2^, *p* = 0.04, and −0.57, *p* = 0.01). A comparison of HRV parameters between the two groups failed to reach statistical significance. Similarly, although an improvement of HRT from the baseline was found only in patients treated with empagliflozin (*p* = 0.01), the latter was not significant in intergroup comparison. Empagliflozin treatment reduced uric acid levels, systolic blood pressure, and body mass index. The authors concluded that the improvement of cardiac sympathetic and parasympathetic nerve activity could somewhat explain the reduction in cardiovascular deaths, in particular, sudden cardiac death caused by fatal arrhythmias [21].

Three main mechanisms may explain the improvement in HRV and HFT after treatment with SGLT2i. The first mechanism is the hemodynamic effect, which is due to the reduced intravascular volume secondary to the osmotic diuresis and natriuresis. The reduction in intravascular volume led to hemodynamic changes which improved cardiac workout and left ventricular function without modification of heart rate, thus suggesting that SGLT2i reduce the reflex sympathetic nerve hyperactivity. Another mechanism is the possible direct inhibition of the myocardium sodium/hydrogen exchanger which may lead to increased mitochondrial function and reduced oxidative stress, thus potentially reducing the onset of arrythmias [22]. The third possible mechanism is the reduced activity of rostral raphe pallidus (rRPa) [23], which physiologically increases the cardiac sympathetic nerve activity and heart rate [24].

## 5. Possible Antiarrhythmic Effects of SGLT2i After AMI

Atrial fibrillation is one of the most frequent arrhythmias in adults [25] and specific risks factors have been recognized [26]. Recent data showed that SGLT2i could have possible antiarrhythmic properties [27].

An extensive body of evidence, confirmed by systematic review and meta-analysis, reported an antiarrhythmic effect of the SGLT2i on specific cardiovascular disease [28,29], but the effect of SGLT2i on cardiac arrhythmias post-myocardial infarction has been studied less. The study by Cesaro et al. investigated the onset of arrhythmias in a sample of diabetic patients hospitalized for STEMI or NSTEMI and treated with PCI [30]. The cohort included 646 patients, divided into two groups based on the use of SGLT2i or not. Authors reported 91 cases of new-onset cardiac arrhythmias during the length of hospitalization: 56 were atrial fibrillation and 35 were ventricular tachycardia/fibrillation. Patients treated with SGLT2i had a lower rate of atrial fibrillation and ventricular tachycardia/fibrillation than non-users (4.5% vs. 9.5% and 1.8% vs. 6.2%, *p* < 0.05). In addition, the use of SGLT2i was associated with a reduction of 65% in the risk of new-onset arrhythmias in the multivariate logistic regression [30].

The association between atrial fibrillation and diabetes is complex and not limited to the presence of obesity. Indeed, inadequate glycemic control and longer duration of diabetes dramatically increase the risk of onset of atrial fibrillation. SGTL1 is expressed in cardiac capillaries and cardiomyocytes, which play a role in glucose transport across the membrane. Despite the low expression of SGLT2 in the cardiac cells, SGLT2i may have an antiarrhythmic effect due to direct and indirect mechanisms. Indeed, SGLT2i could decrease the activity of the sympathetic nervous system and significantly reverse cardiac and anatomic remodeling [31,32]. In addition, SGLT2i regulate sodium and calcium channels and reduce the development of calcium-related arrhythmias [33]. SGLT2i may also have indirect beneficial effects through their natriuretic effect, enhanced ketone oxidation, and body weight loss [34].

## 6. The Effects of SGLT2i on Heart Failure Occurrence After AMI

The SGLT2i have positive effects on all patients with heart failure, independently of left ventricular ejection fraction values [29,35,36]. Further, early treatment helps to delay or reverse cardiac remodeling and prevents progression to symptomatic heart failure [37]. Robust evidence demonstrated that the use of SGLT2i reduced the risk of rehospitalization in patients with chronic heart failure. Indeed, the DELIVER trial showed that dapagliflozin treatment reduced the risk of worsening of heart failure, defined as unplanned hospitalization or emergency visits for heart failure [38]. However, this trial also found that there was no significant difference in cardiovascular mortality between the groups. Thus, a possible bias should be mentioned: the reduction in the risk of the primary composite outcome with Dapagliflozin was primarily attributed to the worsening of heart failure.

The role of SGLT2i on the rate of heart failure rehospitalization after AMI has been recently demonstrated (Table 1). A Chinese study included 961 diabetic patients with AMI treated with PCI during hospitalization [39]. The subjects were stratified into two groups: those who were started on dapagliflozin 10 mg daily and those who were treated with other glucose-lowering drugs. The analysis showed that, after a median follow-up of 540 days, patients treated with SGLT2i had a significantly lower heart failure rate than those treated with other glucose-lowering drugs (*p* < 0.001). In addition, the use of Dapagliflozin reduced the risk of heart failure rehospitalization (HR 0.50, 95%CI 0.30–0.83, *p* < 0.001) [39].

Another study, based on National Health insurance claims data in South Korea, proposed an analysis based on a propensity score matching [40]. The authors revised over 30,000 clinical records and found 938 patients with type 2 diabetes treated PCI for AMI and who received SGLT2i within 14 days after PCI. The patients with or without SGLT2i were matched at a 1:2 ratio. After a median follow-up of 2.1 years, the use of SGLT2i was associated with a lower risk of a composite outcome of hospitalizations for heart failure and all-cause death (7.4% vs. 9.8%, HR 0.68, 95%CI 0.54–0.87, *p* = 0.002) [40].

Similarly, the study by Zhu et al. showed that, during a median of 23 months, patients treated with dapagliflozin had a lower rate of heart failure compared to those treated with other antidiabetic drugs (2.1% vs. 10.4%, *p* < 0.001) [41]. Interestingly, DAPA administration was significantly associated with a reduced risk of HF (*p* = 0.003), non-fatal myocardial infarction (*p* = 0.005), unplanned repeat revascularization (*p* = 0.031), and the composite outcome of major adverse cardiovascular events (*p* = 0.009), but not non-fatal stroke (*p* = 0.072). However, it should be noted that other studies reported no effect of SGLT2i on rehospitalization for heart failure [41]. Indeed, the study by Adel et al. tested the impact of dapagliflozin 10 mg in a double-blind, randomized, controlled clinical trial in a sample of diabetic patients with acute coronary syndrome treated with PCI [42]. The authors noticed no difference between the two groups, but the small sample limited the statistical power, and no hospitalization for heart failure was noted. Similarly, the EMMY trial, which was a double-blind trial, enrolled 476 patients with AMI [43]. The patients were randomized to 10 mg of empagliflozin or placebo, and the medication was started within 72h of PCI. After 26 weeks of follow-up, both groups experienced a decrease in the N-terminal pro-Brain Natriuretic Peptide (NT-proBNP), which was more pronounced in those treated with SGLT2i. In this group, a greater reduction was observed at 12 weeks. Secondary analysis, however, showed that hospitalization for heart failure did not differ significantly between the two groups, as reported in Table 1 [43]. The timing of SGLT2i prescription has no effect on heart failure hospitalization, as reported in the study by Butler et al. (EMPACT-MI trial) [44]. This randomized, double-blind, placebo-controlled trial included 3260 patients treated with empagliflozin started within 14 days after admission for AMI and 3262 subjects treated with placebo. The primary endpoint (composite of hospitalization for heart failure or death from any cause) occurred at a comparable frequency among the two groups: 8.2% of patients treated with SGLT2i and 9.1% of patients in the control group (*p* = 0.21) [44]. However, additional analysis of the same trial revealed that the risk for first HF hospitalization and total HF hospitalizations was significantly lower in the empagliflozin compared with the control group (3.6% vs. 4.7%, HR 0.77 95%CI 0.60–0.98 *p* = 0.031, and 148 vs. 207 events, HR 0.67 95%CI 0.51–0.89 *p* = 0.006, respectively) [45].

The role of SGLT2i in heart failure hospitalization in patients with AMI and treated with PCI is not clear. Indeed, some reported a reduction in the risk of rehospitalization, but others showed no effect. Methodological issues can in part explain the different results because some studies had a small number of subjects while others, albeit with a wider sample, had a lower frequency of older subjects, thus possibly underestimating the incidence of heart failure hospitalization. However, it should be noted that SGLT2i have a consolidated role in the management of chronic heart failure, as reported in the latest guidelines [46]. Indeed, SGLT2i can increase diuresis thanks to their effect on glycosuria and natriuresis [6]. In addition, they modulate the sympathetic nervous system and the reduction in oxidative stress and inflammation [47].

**Table 1:** Studies that tested the role of SGLT2i in heart failure rehospitalization after acute myocardial infarction.

Author	Type of SGLT2i	Timing of SGLT2i Initiation	Sample		Follow-Up	Events		Results
			SGLT2i	Controls		SGLT2i	Non-SGLT2i	
Mao [39]	D	N/A	231	231	540 days	13 (5.6%)	35 (15.2%)	SGLT2i reduced the risk of heart failure rehospitalization
Zhu [41]	D	N/A	141	645	23 months	3 (2.1%)	67 (10.4%)	SGLT2i reduced the risk of heart failure rehospitalization
Adel [42]	E	N/A	45	48	6 months	0 (0.0%)	0 (0.0%)	No effect
von Lewinski [43]	E	Within 72 h from PCI	237	239	26 weeks	3 (1.3%)	4 (1.7%)	No effect
Butler [44]	E	Within 14 days	3260	3262	17.9 months	267 (8.2%) *	298 (9.1%) *	No effect on composite outcome (heart failure and death from any cause)
Hernandez [45]	E	Within 14 days	3260	3262	17.9 months	118 (3.6%) ^#^ 148 (4.5%) ^##^	153 (4.7%) ^#^ 207 (6.3%) ^##^	SGLT2i reduced the risk of HF in patients with left ventricular dysfunction or congestion after acute myocardial infarction
Kwon [40]	N/A	Within 14 days	938	1876	2 years	68 (7.4%) ^###^	166 (9.8%)	SGLT2i reduced the risk of hospitalizations for heart failure

Abbreviation: D, dapagliflozin; E, empagliflozin; PCI, percutaneous coronary intervention; N/A, not applicable. * Composite outcome (heart failure and death from any cause). ^#^ First heart failure hospitalization. ^##^ Total number of heart failure hospitalizations; ^###^ Composite outcome (all-cause death and hospitalizations for heart failure).

## 7. The Effects of SGLT2i on Ventricular Remodeling After AMI

Several studies have examined the effect of SGLT2i on specific echocardiographic parameters after AMI, as reported in Table 2. The study by Wan et al. retrospectively included 423 patients with type 2 diabetes mellitus and AMI treated with PCI. The sample was divided into two groups according to the use of SGLT2i [48]. After six months from discharge, patients treated with SGLT2i had a significantly lower left ventricular remodeling index (3.49 ± 19.71 vs. 7.06 ± 15.15, *p* < 0.05), while non-SGLT2i had a higher frequency of left ventricular remodeling >20%, considered pathologic threshold by the authors and calculated as a 20% or greater increase in left ventricular end-diastole volume between baseline and six-month follow-up [48].

The timing of the prescription of SGLT2i after myocardial infarction seems to be critical, even in non-diabetic patients. Indeed, to test this hypothesis, Abdel Dayem et al. performed a double-blind randomized controlled trial of patients with AMI treated with PCI [49]. The patients, free from diabetes mellitus and with echocardiographic evidence of reduced left ventricular ejection fraction <50%, were randomized to receive either placebo or dapagliflozin 10 mg daily. Follow-up visits were set at 4 and 12 weeks. NT-proBNP was assessed at baseline visit and after 12 weeks, while echocardiographic parameters were collected at baseline and in the two follow-up visits. Patients treated with dapagliflozin had a greater decrease in NT-proBNP levels from baseline to 12 weeks compared to the control group (133 ng/L vs. 89 ng/L, *p* < 0.05). Further, at 12 weeks the left ventricular mass index in the treatment group was less than the control group by 11.46% (95%CI: −19.37 to −3.56, *p* = 0.029) [49]. However, it should be noted that other authors found no significant advantages of early administration of SGLT2i. Indeed, the EMMY study evaluated the effect of empagliflozin on cardiovascular parameters (left ventricular function, left ventricular end-systolic, and end-diastolic diameters) [50]. Specifically, the sample was divided into three groups according to the timing of administration of SGLT2i after PCI: early (<24 h), intermediate (24–48 h), and late (48–72 h). The analysis revealed that SGLT2i treatment, compared to the control group, had a positive effect on the above-mentioned echocardiographic parameters, regardless of the timing of administration [50].

The modification of echocardiographic parameters was also studied with magnetic resonance in the EMPRESS-MI trial [51]. This randomized, double-blind, placebo-controlled, multicenter study enrolled 105 patients with a recent diagnosis of myocardial infarction (≥12 h and <14 days) and left ventricular ejection fraction <45% on magnetic resonance imaging at baseline. The patients, who were randomized to empagliflozin 10 mg daily or placebo, performed a cardiovascular magnetic resonance at baseline and after 24 weeks of treatment. The analysis revealed that treatment with SGLT2i, when compared to placebo, did not significantly reduce the left ventricular end-systolic volume indexed to body surface area (8.3 ± 13.5 mL/m^2^ vs. 7.8 ± 16.3 mL/m^2^). Similarly, the change in the left ventricular end-diastolic volume indexed to the body surface area (0.6 ± 16.3 mL/m^2^ and −0.3 ± 18.7 mL/m^2^), the increase in the left ventricular ejection fraction (9.4 ± 7.5% vs. 8.5 ± 7.4%), and the indexed left atrial volume (37.3 mL/m^2^ vs. 39.2 mL/m^2^) were comparable between the two groups [51].

The effect of SGLT2i on ventricular remodeling in heart failure patients is well known [52], but their impact after AMI is less studied. SGLT2i may contribute to the detrimental effect at the ventricular level after AMI. Different mechanisms have been postulated. Indeed, SGLT2i could reduce inflammation, which is key in ventricular remodeling [53], as demonstrated by the reduction in interleukin-1β secretion and NLRP3 inflammasome activity in macrophages [54,55]. A role in sympathetic nerve activity has been revealed, as previously described. In addition, SGLT2i may also have a positive impact on cardiac fibrosis by reducing fibroblast activation and extracellular matrix remodeling [56].

**Table 2:** Modification of echocardiographic parameters in patients treated with SGLT2i after acute myocardial infarction.

Author	Sample		Follow-Up	Echocardiographic Parameter	Baseline		Post-Treatment		Results
	SGLT2i	Control			SGLT2i	Control	SGLT2i	Control	
Wan [48]	239	184	6 months	LVEF	53.53 ± 10.20	56.25 ± 9.27	2.17 ± 6.66 *	0.55 ± 9.51 *	SGLT2i protects against LVR
				LVEDV	101.92 ± 30.38	98.17 ± 21.83	1.42±18.10 *	6.36 ± 14.75 *	
				LVESV	49.64 ± 26.62	44.44 ± 19.02	−2.16 ± 14.89 *	2.87 ± 16.02 *	
				LVRI	N/A	N/A	3.49 ± 19.71	7.06 ± 15.15	
Dayem [49]	50	50	12 weeks	LVEF	42.70 ± 7.21	43.29 ± 5.23	50.33 ± 7.49	49.84 ± 8.22	Early SGLT2i administration improves cardiac function
				LVEDd	47.62 ± 0.59	46.28 ± 0.88	47.97 ± 0.82	46.85 ± 0.79	
				LVESd	35.76 ± 0.66	35.56 ± 0.74	35.08 ± 0.73	34.61 ± 0.49	
				LV mass	103.34 ± 18.12	101.47 ± 19.50	93.60 ± 21.58	103.53 ± 17.18	
				ePASP	33.4 ± 9.15	32.90 ± 10.31	31.10 ± 11.54	30.71 ± 10.65	
Carberry [51]	51	53	24 weeks	LVESVI	65.6	62.8	57.2	55	SGLT2i had no effect on cardiac volumes and LVEF
				LVEDVI	97.8	97.6	98.3	97.3	
				LVEF	33.4	36	42.7	44.4	
				LAVI	34.3	36.2	37.3	39.2	
				LVMI	62.2	59.1	52.2	50.4	

Abbreviations: LVEF, left ventricular ejection fraction; LVEDV, left ventricular end-diastolic volume; LVESV, left ventricular end-systolic volume; LVRI, left ventricular remodeling index; LVMI, left ventricular mass index; LAVI, left atrial volume index; LVEDd, left ventricular end-diastolic diameter; LVESd, left ventricular end-systolic diameter; LV mass, left ventricular mass; ePASP, estimated pulmonary artery systolic pressure; LVESVI: left ventricular end-systolic volume; LVEDVI: indexed left ventricular end-diastolic volume; N/A, not applicable. * Difference between echocardiographic parameter at the end of the follow-up and baseline echocardiographic parameter.

## 8. The Possible Protective Role of SGLT2i on Acute Kidney Injury After AMI

The kidney has a pivotal role in glucose homeostasis due to its capacity to reabsorb the glucose filtered into the glomerular filtrate. In healthy individuals, no glucose is usually present in the urine because all the glucose filtered is reabsorbed in the kidney, and glycosuria occurs when blood glucose exceeds 180 mg/dL [57]. SGLT2 is located in the proximal renal tubule, allowing kidney cells to reabsorb the filtered glucose. Inhibitors of SGLT2 increase glucose excretion in the urine by lowering the renal threshold for glucose excretion independently from insulin.

Recent studies highlighted the importance of SGLT2i after AMI to reduce the risk of acute kidney injury (AKI). Indeed, the administration of iodinated radiocontrast material during PCI increased the risk of developing AKI, which is somewhat modified by additional factors such as age, baseline estimated glomerular filtration rate, and use of contrast agent [58]. This condition may lead to adverse outcomes, such as increased in-hospital and long-term mortality [59]. The study by Cai et al. retrospectively analyzed a cohort of 1839 patients with AMI treated with PCI [60]. The sample was divided into two groups (dapagliflozin users vs. control group), whose baseline characteristics were balanced through a propensity score matching analysis. The primary outcome was the occurrence of AKI 7 days after PCI. The frequency of AKI was lower in the treatment group (15.1% vs. 19.1%), and the multivariate regression analysis, adjusted for confounders, revealed that treatment with SGLT2i was a protective factor for AKI development (OR 0.66, 95%CI 0.44–0.97, *p* = 0.036). Similar results have been obtained in a single-center case–control study, which reported that diabetic patients treated with SGLT2i had a lower risk of developing contrast-induced AKI (OR 0.86, 95%CI 0.76–0.98, *p* = 0.028) [61]. A recent meta-analysis summarized the efficacy of SGLT2i in reducing the risk of AKI (RR 0.46; 95%CI 0.32–0.67, *p* < 0.0001) [62]. Clinical and experimental data have shown that the use of SGLT2i improves kidney function through multiple mechanisms. Indeed, SGLT2i may improve renal oxygenation by activating antifibrotic and anti-inflammatory pathways [63]. Further, dapagliflozin may inhibit hypoxia-inducible factor-1α/human epididymis secretory protein 4/nuclear factor-κB signaling, thus reducing the risk of contrast-induced AKI [64] and reducing oxidative stress, fibrosis, and inflammatory markers [65]. Of course, a clear, direct effect of diuretic osmosis has also been demonstrated. Indeed, the increased diuresis decreases the plasma volume, which activates the tubule-glomerular feedback. The latter may increase sodium reabsorption, cause vasoconstriction of afferent arterioles, and reduce the renin-angiotensin system by decreasing intraglomerular pressure [66].

## 9. Conclusions

SGLT2i have a critical role in the management of heart failure and are not only related to the presence of diabetes. Current data support the concept that patients improve the clinical control of chronic heart failure and reduce the risk of exacerbation. In this review, we showed that SGLT2i seem to have additional effects in patients with AMI treated with PCI. The overall benefits of early treatment may reduce the risk of the onset of specific complications, such as arrhythmias and ventricular remodeling, which often develop after AMI. However, the benefits for other specific diseases need to be clarified. To address critical knowledge gaps and better understand the mechanisms underlying the beneficial effects of SGLT2i in heart failure, future research should be clearly outlined.

## Data Availability

Not applicable.
